# Supplementary material for: Isoniazid preventive therapy completion between July-September 2019: A comparison across HIV differentiated service delivery models in Uganda
Source: PLoS One. 2024 Jan 2;19(1):e0296239. doi: 10.1371/journal.pone.0296239 (PMC10760732; doi:10.1371/journal.pone.0296239)
Supplement: Attachment — Submitted filename: Response to Reviewers.docx [file pone.0296239.s003.docx]

**Response to reviewers**

**Journal Requirements:**

When submitting your revision, we need you to address these additional requirements.

1. Please ensure that your manuscript meets PLOS ONE's style requirements, including those for file naming.

Response: Manuscript has been revised to address all PLOS ONE style requirements.

2. Please include a complete copy of PLOS’ questionnaire on inclusivity in global research in your revised manuscript. Our policy for research in this area aims to improve transparency in the reporting of research performed outside of researchers’ own country or community. The policy applies to researchers who have travelled to a different country to conduct research, research with Indigenous populations or their lands, and research on cultural artefacts. The questionnaire can also be requested at the journal’s discretion for any other submissions, even if these conditions are not met.  Please find more information on the policy and a link to download a blank copy of the questionnaire here: <https://journals.plos.org/plosone/s/best-practices-in-research-reporting>. Please upload a completed version of your questionnaire as Supporting Information when you resubmit your manuscript.

Response: A copy of PLOS questionnaire on inclusivity in global research has been attached as supporting information.

3. In the ethics statement in the manuscript and in the online submission form, please provide additional information about the patient records/samples used in your retrospective study. Specifically, please ensure that you have discussed whether all data/samples were fully anonymized before you accessed them and/or whether the IRB or ethics committee waived the requirement for informed consent. If patients provided informed written consent to have data/samples from their medical records used in research, please include this information.

Response: The ethics section has been revised as follows: “The study was registered by the Uganda National Council for Science & Technology (HS1228ES). A waiver to review and extract data from existing medical records was obtained from the TASO-Research Ethics Committee (TASOREC/004/2020-UG-REC-009) and the Columbia University Irving Medical Center IRB (IRB-AAAS9111). Furthermore, we obtained administrative clearance from the Ugandan Ministry of Health, the district health officers, the executive director of TASO, and the In-charges of the participating facilities. Data anonymization was implemented prior to data extraction.”

4. Your ethics statement should only appear in the Methods section of your manuscript. If your ethics statement is written in any section besides the Methods, please move it to the Methods section and delete it from any other section. Please ensure that your ethics statement is included in your manuscript, as the ethics statement entered into the online submission form will not be published alongside your manuscript.

Response: The ethics statement has been revised as requested and moved to the methods section.

**Reviewers' comments:**

**Reviewer #1:**

Dear PLOS One Team of editorials, thank you for the chance given to me to review “Completion of Isoniazid Preventive Therapy among People Living with HIV on ART across Differentiated Service Delivery (DSD) Models in Uganda”. The research article has its own importance for the improving the health of PLWH in particular and the general public in general. The following are my comments.

Response: Thank you for acknowledging the importance of this research article.

1. General Comments

The justification to conduct the study was not strong especially in the abstract section.
Why Isoniazid preventive therapy? Is PLWH are significant in number? Why across differentiated service? Why not on the particular service? IS that across the nations? IS the program new?

Response: We have strengthened the rationale for this study in the Abstract (Lines 34-35) and the Introduction section (lines 66-78). The recommended regimen for TB prevention among PLHIV in Uganda is IPT delivered via DSD models, as those have shown to be effective in delivering ART to PLHIV. Because little is known about the impact of delivering IPT through different DSD models, we determined IPT completion and associated factors across DSD models.

Use of correct words, sentences, grammar, paragraph and the whole sections content should be well revised.

Response: Thanks for noting this. The authors conducted a careful review and revised the current manuscript for grammatical and spelling errors.

Avoid use of abbreviations in the abstract section.

Response: We minimized use of abbreviations to the extent possible and ensured that every abbreviation is defined on first use, with the exception of HIV which is in the English dictionary.

Use confidence interval whenever describing the percentage of the main objective.

Response: We updated the manuscript to include confidence intervals.

Address the consistency from the beginning to the end of the article.

Response: Consistency has been addressed in the revised manuscript.

Add in to key words. E.g. Uganda.

Response: Uganda has been added as a key word.

2. Specific Comments

• Make sure that all the elements of the background section are fulfilled. Describe what is known and unknown and what gaps you want to fill by your study.

Response: Thank you for pointing this out. We have revised the introduction and described what is known and unknown, and explicitly explained the gap that our study fills (lines 92-108).

• In the result of the abstract section it is not mandatory to report both the OR and the P value since AOR is stronger than the P value.

Response: We agree with the reviewer and have removed p-values from the abstract where aRR is reported.

• It is use of secondary data? Is the data complete? How Did you assess the validity and reliability of the study? Are the data sharing criteria met? Is that freely accessible or accessible per request? What are the steps taken hence?

Response: Yes, the study used secondary data with some data missing on some variables. We used multiple imputation to handle missing data. All data used in the analysis has been made available as supplementary material.

• Did the contents of all sections have well ensured entailing all the contents? E.g. did the background, methods, results and discussions contains what it has intended to contain?

Response: After making some adjustments, we confirm that all sections contain the intended relevant details.

• Use correct tense, grammar, sentence, language, paraphrase, consistency…etc needs meticulous correction.

Response: Thank you for pointing this out. We have conducted a thorough review and corrected grammatical and spelling errors.

• Which type of data should be presented textually, by chart and by table?

Response: Because we were interested in stratifying our results by DSD models, we chose to present the results in tables with summary text.

• Revisit the numbers, totals and the statistics in general.

Response: We have conducted a thorough review to ensure that all numbers, totals, and statistics are accurate.

**Reviewer #2:**

Thank you for your submission. Although the article presents a interesting finding, its applicability, originality and contribution to existing knowledge base is questionable. A recent publication from LMIC settings include:

Mukumbwa-Mwenechanya M, Mubiana M, Somwe P, Simwenda M, Zyongwe N, Kalumkumya E, Mwango L, Rabkin M, Mpasela F, Chungu F, Mwanza F. Integrating Isoniazid Preventive Therapy into the Fast-Track HIV Treatment Model in Urban Zambia: A Proof-of-Concept Pilot Project. medRxiv. 2022 Jan 1.
Guthrie T, Muheki C, Rosen S, Kanoowe S, Lagony S, Greener R, Miot J, Balidawa H, Kiggundu J, Calnan J, Dejene S. Similar costs and outcomes for differentiated service delivery models for HIV treatment in Uganda. medRxiv. 2021 Jan 1.

Response: Thank you for highlighting these two recent preprint publications. We believe that these studies do not dimmish from the applicability, originality and contribution of our article to the existing knowledge.

The study from Zambia, which was conducted around the same time as our study, is relevant and have added some of its findings to our Discussion section. However, please note that the Zambia study only looked at integrating IPT into the FT model, whereas our study examined integration of IPT across multiple DSD models, including community- and facility-based models. The study from Uganda mentioned above, measured cost per patient and cost per patient virally suppressed on ART in Uganda across five DSD models but did not consider the integration of IPT into these DSD models.
