# Supplementary material for: Isoniazid preventive therapy completion between July-September 2019: A comparison across HIV differentiated service delivery models in Uganda
Source: PLoS One. 2024 Jan 2;19(1):e0296239. doi: 10.1371/journal.pone.0296239 (PMC10760732; doi:10.1371/journal.pone.0296239)
Supplement: Attachment — Submitted filename: Response to Reviewers.docx [file pone.0296239.s005.docx]

We thank the Reviewers for their thoughtful comments regarding our manuscript and the opportunity to revise our manuscript. We have revised the manuscript, which we believe has been substantially improved by incorporating the changes recommended by the Reviewers. Below, we provide a point-by-point response to the Reviewers’ comments and reference corresponding line numbers in the text with these changes.

a. The title should be changed in a way that best suit it and add a time period.

**Response**: Thank you for this excellent suggestion. The title has been revised and now reads as follows “Isoniazid Preventive Therapy Completion between July-September 2019: A Comparison across HIV Differentiated Service Delivery Models in Uganda”

b. It is comparison between the (a) the old and (b) new model of Isoniazid delivery. Hence, the method should be comparative study design. The study had assessed the status of completion of the Isoniazid prophylaxis and the findings suggest that the old model had better completion due to known reasons. Hence, what is new? It is an implementation study and what type of implementation study is that?

**Response**: Dear reviewer, thank you for this suggestion. While we are citing past known IPT completion rates from other national explorations of old models of IPT delivery, we are not directly conducting a pre and post comparison of IPT completion rates. In this paper, we explore how IPT completion differed across various approaches of IPT delivery, known as differentiated service delivery (DSD) models. We updated the title to clarify that.

DSD models in Uganda have been implemented over the last 4-11 years depending on the implementer. However, other services like IPT were not layered into them but were left for facility-based individual models. The government eventually layered IPT within the different DSD models and we set out to evaluate and compare IPT completion rates and associated factors across DSD models. Please note that individuals in the community DSD models tend to be those who have been on ART longer because of the initial tendency to move only stable clients to community models.

Lastly, we did not conduct an implementation study but simply evaluated the new government strategy for IPT delivery that is layered within DSD models. The government of Uganda continues to layer many new innovations within DSD models; thus, evaluation of such novel ideas is paramount to guide the improvement of strategy to deliver them.

c. You didn’t include the clinical characteristics like viral load since its completion study. What about the status of completeness of data, data quality and how you had treated the data.

**Response**: Thank you for this observation. Data on viral load was not extracted and hence could not be analysed; this has been added as a limitation (lines 279-281). Regarding data completeness, data quality and strategies to address, where data were missing from the electronic data system, we cross-checked with patient care cards or IPT registers and abstracted data with the help of a digital data abstraction tool installed on tablet computers (see lines 126-127). To account for missing data, multiple imputations using a multivariate normal model were used (see lines 141-142). In addition, we acknowledged and reported missing data for each variable in the tables.

d. It is senses and you have conducted regression analysis which is Poisson. Is that the recommended solution? If it is even survey, is the case to variable ratio fits the model?

**Response**: Thank you for this comment. In this paper, we wanted to estimate and compare completion rates (estimated as risk ratio) across DSD models. A modified Poisson fitted by specifying robust standard errors is an appropriate and commonly used strategy, although other models like log-binomial can be used. Naimi and Whitcomb (2020) give a clear discussion and justification for use of these models; their reference has been included in the paper (lines 138 and 347).

e. All the sections of the manuscript should entail what scientifically it intends to entail.

**Response**: We reviewed the manuscript and ensured that each section entails the appropriate details as guided.

f. The Manuscript should be geared with the format of the PLOS ONE and language and statistics should be revisited and appropriate analysis and assumptions should be used and reported.

**Response:** We carefully reviewed the manuscript (including the statistics) to make sure it conforms to the required format, and are happy to work with the editor further to ensure full compliance.
