# Supplementary material for: Isoniazid preventive therapy completion between July-September 2019: A comparison across HIV differentiated service delivery models in Uganda
Source: PLoS One. 2024 Jan 2;19(1):e0296239. doi: 10.1371/journal.pone.0296239 (PMC10760732; doi:10.1371/journal.pone.0296239)
Supplement: Attachment — Submitted filename: Response to Reviewers.docx [file pone.0296239.s006.docx]

We thank the editor for the thoughtful comment regarding our manuscript and the opportunity to revise our manuscript. We have revised the manuscript to address this comment. Below, we provide a response to the editor’s comment and reference corresponding line numbers in the text with these changes.

a. In the previous review I questioned the multiple imputation for categorical variables using multivariate normal. Authors responded saying it was done using dummy variables for all categorical variables. I believe this is impossible to do. Because, all data generated by multivariate normal process for missing values are in continuous scales, not in dummy coded scales as required by categorical variables. Please include a clarification for this within the manuscript with a suitable reference.

**Response**: Thank you for this excellent observation. We have revised the results using multiple imputation chained equations (MICE) which enables us to impute missing data for both categorical and continuous variables (instead of using a multivariate normal model) (see lines 140-141). However, we noticed a very small difference in the results and in most cases the estimates remained the same (see lines 193-196, and Tables 2 and 3).
