# Supplementary material for: Isoniazid preventive therapy completion between July-September 2019: A comparison across HIV differentiated service delivery models in Uganda
Source: PLoS One. 2024 Jan 2;19(1):e0296239. doi: 10.1371/journal.pone.0296239 (PMC10760732; doi:10.1371/journal.pone.0296239)
Supplement: Attachment — Submitted filename: Response to Reviewers.docx [file pone.0296239.s007.docx]

Dear Editor,

We thank the Reviewers for their thoughtful comments regarding our manuscript and the opportunity to revise our manuscript further. Unfortunately, the wrong document was uploaded for Reviewer 1 so we were not able to address their concerns. However, we have revised the manuscript, which we believe has been substantially improved by incorporating the changes recommended by Reviewer 3. Below, we provide a point-by-point response to the Reviewer’s comments and reference corresponding line numbers in the text with these changes.

**Reviewer #3:**

The authors have explored a critical topic on DSD and completion rates of IPT. This area of study is important in informing efficient IPT delivery approaches among this group that has high burden of TB. The article makes an important contribution. One of the major concerns about the article is missingness in data. The variable age, had close to 50% missingness, this level of missingness is worrying and going by this, missingness is likely to have also affected other variables. The authors mention it in the limitation section but do not divulge the extent of missingness, its effect on the estimates reported and the caution readers should apply when interpreting their results. Missingness and how it was addressed should be included methods section as part of the analysis approach used. Data imputation can be done to address missingness but there are limits to imputation. This is the greatest weakness of this article.

Response: Thanks so much for raising this very important issue. We agree that having a high proportion of missing data in the Age at IPT initiation variable, could have affected the results even with multiple imputation. We noted that we had not produced results for complete case analysis which was an error and could have prevented one from observing the effect of missing data. We updated the results to include complete case analysis and are now considering the results from the multiple imputation as sensitivity analysis. We observe that adjusted results from multiple imputation generally did not differ from those from complete case analysis when looking at factors associated with IPT completion in each DSD model (Table 3). However, when comparing IPT completion across DSD models (Table 2), there is an association with CCLAD and CDDP in the complete case analysis, but the effect disappears with multiple imputation. When analyzing the missing age with DSD model, we noted that all participants in CCLAD had data available on age, which could explain the difference in observed results. Revisions have been made to the manuscript to clarify the effect of missing data. See lines 143-150; Lines 187-192; Table 2; and Table 3.

1. The analysis evaluates IPT completion among patients who initiated IPT between July-Sep 2019, IPT is taken for 6-9 months then the outcome is evaluated. This is not a cross-sectional study. Kindly revise.

Response: Thank you for this observation. We have revised the study design as follows “This was a retrospective study with review of electronic medical records and patient registers for PLHIV who initiated IPT during the period of July-September 2019.” (Lines 110-111)

2. In the methods section the authors have a section that describes conduct of census yet this was a data review. This is confusing, kindly clarify

Response: This has been clarified as data review by revising the statement to read as follows “We conducted a medical records review of all PLHIV who had initiated IPT at the three selected health facilities during July-September 2019.” (Line 120-121)

3. Did the investigators evaluate IPT completion rates by duration on ART? Include results.

Response: Yes, we investigated the effect of duration on ART at time of ITP initiation. However, this was not significantly associated with IPT completion in all DSD models except CCLAD where it’s only significant in unadjusted analysis. After adjusting for other factors, it became insignificant both for complete case analysis and with multiple imputation. Results are now shown in Table 3 for the CCLAD model.

4. I seem to have missed IPT completion rates by gender. Was this analysed?

Response: Thank you for this observation. We have now added analysis of completion rate by gender in each DSD model, and its effect shows up in FBG and CCLAD models. Results are now presented in Table 3 and in lines 204-205.

5. One portion of the results stands out. The CCLAD unadjusted RR is not significant with a high p-value (0.996), upon adjustment this turns highly significant (p<0.001) which is not impossible but rather unusual. Review this result and confirm.

Response: Thank you for catching this. We revisited the multiple imputation method using multiple imputation chained equations (MICE) on factor variables instead of using the multivariate normal models on dummy variables (derived from the factor variables) which we had used previously. With the MICE approach, the p-values for unadjusted and multiple imputation results are now consistent (see Table 2).

6. The assertion that “community based DSD models are more person-centered for IPT” is not accurate because the data despite showing higher completion rates does not support this statement. The authors do not have survey data neither do they present qualitative data or any other data that supports the assertion or demonstrates that the facility delivery models are less person-centered. Whereas, I fully agree with their postulation that a reduction in travel cost and time may have driven higher uptake and completion of IPT due to convenience offered by the model, this does not necessarily equate to person-centered care. Kindly revise.

Response: Thank you for noting that; the statement has been deleted from the Discussion section.
