# Supplementary material for: Isoniazid preventive therapy completion between July-September 2019: A comparison across HIV differentiated service delivery models in Uganda
Source: PLoS One. 2024 Jan 2;19(1):e0296239. doi: 10.1371/journal.pone.0296239 (PMC10760732; doi:10.1371/journal.pone.0296239)
Supplement: Attachment — Submitted filename: Comments to author_IPT completion rates.docx [file pone.0296239.s008.docx]

The manuscript explores IPT completion rates (and related factors) in light of various DSD models in eastern Uganda. The overall sample size is adequate though with significant missing variables – to which the authors try to address. Some sub-analyses also end up being carried out on fewer numbers due to the many variable points. As it has gone through a round of review process, I would just point out a few areas to further improve the manuscript

1. Line 68: one in three has hyphens in between
2. Lines 75-6: the sentence IPT is ‘currently’ the most common form of TPT in resource-limited settings may be intriguing at the time of publication. Shorter TPT regimens are increasingly being used even in resource-limited settings. Thus, either back this sentence with a reference or change tense to reflect past or let it refer to the bulk of PLHIV already in care.
3. Line 85: change ‘recommend’ to ‘recommended’, as the 2014 Uganda IPT guidelines may have been surpassed by revisions.
4. Lines 137-8: The poisson model estimated the RRs by adjusting for multiple variables. Was collinearity considered between ART duration, treatment line and regimen? Was it tested and adjusted appropriately? E.g., the longer one is on ART the likelihood of moving to 2^nd^ and 3^rd^ treatment lines. Equally, guidelines indicate that first lines be DTG-based (an INSTI) leading to potential relationship between treatment line and regimen.
5. Under tables 1 and 3, include the DSD acronyms in full.
6. Line 219 in discussion, also in results table 3 - IPT completion at facility-based models differed between sites: What could have been the likely reason of the consistently lower IPT completion in TASO Soroti facility models? Even an intelligent speculation would suffice in the discussion.
7. Line 296: Delete the word ‘concrete’ in …provide concrete evidence…this is superfluous in this context.

*End*

Thank you
