# Supplementary material for: Isoniazid preventive therapy completion between July-September 2019: A comparison across HIV differentiated service delivery models in Uganda
Source: PLoS One. 2024 Jan 2;19(1):e0296239. doi: 10.1371/journal.pone.0296239 (PMC10760732; doi:10.1371/journal.pone.0296239)
Supplement: Attachment — Submitted filename: Response to Reviewers.docx [file pone.0296239.s009.docx]

Dear Editor,

We thank the Reviewers for their thoughtful comments regarding our manuscript and the opportunity to revise our manuscript further. Below, we provide a point-by-point response to the Reviewer’s comments and reference corresponding line numbers in the text with these changes.

**Reviewer #4:**

Abstract line 56-58: How were clients allocated to various DSD models. Could client allocation have influenced selection of patients with higher probability of getting certain TPT outcomes? Are there possible limitations of the study kindly include these in the abstract.

Response: Thank you for your comment. Stable clients, who are virally suppressed are allocated to three models namely, CCLAD, CDDP and FTDR while unstable clients (virally not suppressed) are allocated to FBIM and FBG. Additional clarity on how patients were allocated to these different models was added to the Methods section (lines 83-85).

We agree that patient allocation could have influenced higher IPT completion rates in certain DSD models and added the following sentence to the discussion “It is possible that the allocation of stable clients to community-based models could explain the higher rates of IPT completion among clients in these DSD models” (Lines 259-260). We also added this as a potential limitation (lines 304-307).

Study limitations are outlined in the Discussion section (Lines 302-311). However, due to the limited word count for the abstract, we could not include these limitations in the abstract.

• Line 66-67: Good information on ART coverage- however you need to quantify this as a percege relative to the need to give the reader a sense of how high ART coverage as you highlight TB as a major problem among PLHIV

Response: Thank you for pointing this out. We have revised as guided as seen in the quote “Despite the impressive scale-up of HIV treatment, with 61.5% (23.3/37.9 million) people living with HIV (PLHIV) in 2018 receiving antiretroviral therapy (ART) at the end of 2018, TB remains the leading cause of death among people living with HIV (PLHIV), accounting for approximately one-in-three AIDS-related deaths” (Lines 66-69)

• Line 118 need to highlight what proportion of the clients were excluded based on missing data on ART initiation

Response. Thank you for this comment. A proportion with missing data on ART initiation has been added (line 119).

• Line 136-138- was the Poisson model adjusted for ART treatment adherence? This could be a potential source of bias in allocation to different DSD models with patients in facility models likely to less adherent to TPT.

Response. Thank you very much for this important observation. Indeed, we would expect patients with better adherence to ART to also have better adherence to IPT, and hence potentially higher IPT completion rate. Unfortunately, data on ART adherence was not readily available and so was not extracted. This has been added to the limitations (Line 308).

• Line 157-164, consider being consistent in reporting your results. Include numerator and percentage for all proportions we note that this is provided for some and not for others.

Response. Thank you! We have now included both numbers (numerators) and percentages as appropriate (Lines 159-168).

• Check for punctuations and spacing across the document

Response: This has been addressed, thank you!

• Line 161, 27.6% does not constitute the majority but rather the largest proportion. You may want to think through this.

Response: Thank you for your suggestion. This has been addressed by using largest proportion instead of majority (line 164).

• Line 190: What about adjustment for ART adherence? This needs to be included in the model as it is a potential source of bias

Response: Again, thank you for this observation. Like mentioned above, we did not extract data on ART adherence and this is pointed out in the limitations (line 308).

• Line 285-292: One major potential source of bias that needs to be highlighted in this study is the fact that in HIV programs patients who are stable on ART and more likely to adhere to HIV treatment and TPT are selected into community DSD models while those who are unstable and less adherent to treatment receive care in facility-based models to ensure that they benefit from additional health care worker support. Thus, the poorer outcomes in facility DSD models may be attributable to selection bias.

Response: We agree with your observation and suggestion. We have included this as a limitation due to potential selection bias (Lines 304-305).

• Line 126 We also need to have a clear determination of how TPT completion was defined. How did the study team verify TPT completion in community-based DSD models given that TPT was dispensed for longer periods? Was dispensing of the last doses of TPT considered completion (as stated in line 126?). What are chances that some clients on DSD were misclassified as having completed TPT when indeed they did not do so? Include in your manuscript how completion was verified

Response: This study was a retrospective review of clients’ medical records and therefore we were not able to verify the IPT completion status. The following statement has been included among the limitations “Fourth, we could not verify IPT completion and we relied on the medical record systems where the completion status was documented.” (Lines 310-311).

**Reviewer #5:**

The manuscript explores IPT completion rates (and related factors) in light of various DSD models in eastern Uganda. The overall sample size is adequate though with significant missing variables – to which the authors try to address. Some sub-analyses also end up being carried out on fewer numbers due to the many variable points. As it has gone through a round of review process, I would just point out a few areas to further improve the manuscript
1. Line 68: one in three has hyphens in between

Response: Done, thank you! (Line 68).

2. Lines 75-6: the sentence IPT is ‘currently’ the most common form of TPT in resource-limited settings may be intriguing at the time of publication. Shorter TPT regimens are increasingly being used even in resource-limited settings. Thus, either back this sentence with a reference or change tense to reflect past or let it refer to the bulk of PLHIV already in care.

Response: Agreed with the reviewer. We dropped “currently” and revised the sentence to “Isoniazid preventive therapy (IPT), which involves daily administration of isoniazid and vitamin B6 for six months, was the most common TPT in resource-limited settings at the time of this study” (Lines 74-76).

3. Line 85: change ‘recommend’ to ‘recommended’, as the 2014 Uganda IPT guidelines may have been surpassed by revisions.

Response: Done, thank you! (Line 86).

4. Lines 137-8: The poisson model estimated the RRs by adjusting for multiple variables. Was collinearity considered between ART duration, treatment line and regimen? Was it tested and adjusted appropriately? E.g., the longer one is on ART the likelihood of moving to 2nd and 3rd treatment lines. Equally, guidelines indicate that first lines be DTG-based (an INSTI) leading to potential relationship between treatment line and regimen.

Response: Thank you for pointing out this oversight of not reporting multicollinearity tests in the manuscript. We used variance inflation factor (VIF) to test for multicollinearity. For all our final fits for which we report adjusted estimates, VIF values were less than 3 indicating no issues for multicollinearity. This has been clarified by adding the following sentence under the data analysis section “Variance inflation factor (VIF) was used to test for multicollinearity in independent variables (Line 145). Also, results on multicollinearity have been added to the results section (Lines 183-184 and Lines 196-197).

5. Under tables 1 and 3, include the DSD acronyms in full.

Response: Done, thank you! (Tables 1 and 3).

6. Line 219 in discussion, also in results table 3 - IPT completion at facility-based models differed between sites: What could have been the likely reason of the consistently lower IPT completion in TASO Soroti facility models? Even an intelligent speculation would suffice in the discussion.

Response: Around the study time, TASO Soroti experienced episodes of stock-out of IPT yet several clients were already initiated on it. This, partly explain the comparatively lower IPT completion rate, especially among individuals in the facility models. By design, unlike those in the community model, PLHIV at facility-based model receive only one to two months of ART and IPT refills due to their being unstable on ART. Therefore, during this period of frequent stock-out of IPT, PLHIV at TASO Soroti facility were disadvantaged whereby several of them could not get IPT drug refills. Secondly, the poorer IPT completion rate at TASO Soroti could have been due to the fact that PLHIV at facility comprise of the most unstable groups with poorer adherence to ART. A paragraph has been added to the discussions (Lines 277-286)

7. Line 296: Delete the word ‘concrete’ in …provide concrete evidence…this is superfluous in this context.

Response: Done, thank you! (Line 318).
