# Supplementary material for: Isoniazid preventive therapy completion between July-September 2019: A comparison across HIV differentiated service delivery models in Uganda
Source: PLoS One. 2024 Jan 2;19(1):e0296239. doi: 10.1371/journal.pone.0296239 (PMC10760732; doi:10.1371/journal.pone.0296239)
Supplement: Attachment — Submitted filename: Response to Reviewers.docx [file pone.0296239.s010.docx]

Dear Editor,

We thank the Reviewers for their thoughtful comments regarding our manuscript and the opportunity to revise our manuscript further. We have revised the manuscript to respond to comments from Reviewers #6 and #7, and we believe it been substantially improved by incorporating the changes recommended by these Reviewers. Below, we provide a point-by-point response to the Reviewers’ comments and reference corresponding line numbers in the text with these changes.

**Reviewer #6:**

Introduction
Line 85.
Kindly quote the 2018 updated guidelines on TB preventive therapy which were in use at the time this study was carried out.

Response: Thank you for this suggestion. Revision is made to quote the 2018 guidelines. Line 111.

Move the description about differentiated service delivery models to the study setting section.

Response: The description about differentiated service delivery models has been moved to study setting section as guided. Lines106-116.

Methods
Study Setting and Population
The study setting and population is inadequately described
The description about differentiated service delivery models and patients eligible for each should be under the study setting not in the introduction

Response: The study setting and population section has been extended by transferring the description about differentiated service delivery models into this section as guided. Lines106-116

Authors should then describe the criteria-if any for initiating patients on IPT, the number of clinic follow-up visits conducted while on IPT-if any, the number of months of IPT dispensed at each clinic refill visit for each DSD model.

Response: Thank you for pointing these out. We have included the following in our revised manuscript. “To be eligible for IPT, a client must be living with HIV and be on ART for any duration. Those who had active pulmonary TB disease were not eligible for IPT initiation. The number of clinic follow-up visits and months of IPT dispensed at each visit varied by DSD models. Clinically stable clients in the community-based DSD models received multi-month (3 or more months) of synchronized IPT and ART refill per visit, while clinically unstable clients in the facility-based DSD models received 1-2 months of synchronized IPT and ART refills per visit. To complete TPT, clients in the community-based DSD models required only two clinic visits, while those in the facility-based models required 3-6 clinic visits within the 6-9 months of IPT initiation”. Lines 119-126.

Study Outcome: How was the main study outcome “TPT completion” measured? Was it the same across all study groups?

Response: TPT completion was measured as a proportion of PLHIV who completed the course of isoniazid treatment within 6-9 months among those who initiated TPT. We have clarified the definition of TPT completion as shown in lines 142-143.

Results
Authors should present only the results. Text explaining the results e.g., Lines 189-191 should be moved to the discussion.

Response: The text explaining results has been transferred to the discussion. Lines 318-319.

Lines 184 and 196, read “there were no issues of collinearity”. Authors should consider changing to ….”there was no collinearity.”

Response: This has been revised as guided. Lines 196 & 206

Line 198-199 read….. “results from the complete case analysis are similar to those from multiple imputation, which implies that the latter method did not produce biased estimates”. Did the did the authors mean the former method? i.e. that the complete case analysis did not produce biased estimates?

Response: Thanks, yes, the statement refers to complete case analysis, the former method and this has been corrected. Line 208.

Authors should consider including some results on common reasons for IPT non- completion since this data was collected – according to lines 128 and 129 in the data collection section

Response: Results on reasons for not completing IPT are now provided. Lines 184-186.

In the discussion section, the authors explained that IPT completion was lower in facility-based models due to episodes of drug stock outs. Was a sensitivity analysis conducted exclude patients affected by the stock out period to check if treatment completion at health facilities still remained lower? i.e. to check that the authors are really measuring IPT adherence not IPT drug availability?

Response: We acknowledge that a sensitivity analysis of drug stock out would help improve our results on IPT completion. However, note that we only obtained qualitative data on stock out and so the sensitivity analysis could not be performed. Secondly, we added further detail write up reflecting the fact that the stock out affected everyone but differentially impacted those in the facility-based designs due to the inherent designs of DSD models which enabled PLHIV in the community-based DSD models to require only 1-2 IPT refills to complete their TPT doses while those in the facility-based model required 3-6 IPT refills.

**Reviewer #7:**

Dear authors: I am reviewing this manuscript for the first time and I appreciate the changes made based on the first round of revisions but I think there are a few more items to revise before publication:

Response: Thank you for taking time to review our manuscript and for your valuable comments.

The IPT completion is very very high across all models. This systematic review PMID 34492003 and this other one PMID 27522233 find much lower proportions. How is it so high in your study setting, even for facility based delivery models? Please provide some additional information to understand this in the context of other studies. This should be explained in the study context and in the discussion.

Response: Thank you so much for these very useful citations on latent TB cascade of care outcomes. We have used these citations to contextualize our findings and enrich our discussion. Lines 251-254

Do we know how many were eligible and thus can include the completion out of the eligible and not only among those who started?

Response: Thank you for your thoughts on estimating completion out of those eligible for IPT. However, the aim of this paper was to estimate completion among those that initiated IPT and thus we restricted data extraction to those who initiated IPT and not those who were eligible for IPT.

I missed the definition of IPT completion in the methods. Was it by number of doses picked up? Was there a range?

Response: IPT completion was measured as a proportion of PLHIV who completed the course of IPT within 6-9 months among those who initiated IPT. This has been clarified in the paper. Lines 142-143

Figure 2 should include confidence intervals and, if used, other statistical tests to compare the completion among hte models.

Response: Figure 2 has been edited to include confidence intervals and the test statistic used.

Please reduce the use of acronyms as it makes it difficult to read. I suggest to keep only the very known ones (HIV, ARV, IPT) but not for each model, each study site, etc.

Response: We have minimized abbreviations as much as possible. We now state site names in full everywhere. However, because the types of DSD models are frequently mentioned in this paper including in the abstract, are well explained in Figure 1, and given in the list of abbreviations section, we have left these abbreviations in the manuscript.
